# Non-Specific dsRNA-Mediated Antiviral Response in the Honey Bee

**DOI:** 10.1371/journal.pone.0077263

**Published:** 2013-10-10

**Authors:** Michelle L. Flenniken, Raul Andino

**Affiliations:** 1 Department of Microbiology and Immunology, University of California San Francisco, San Francisco, California, United States of America; 2 Department of Plant Sciences and Plant Pathology, Montana State University, Bozeman, Montana, United States of America; Ghent University, Belgium

## Abstract

Honey bees are essential pollinators of numerous agricultural crops. Since 2006, honey bee populations have suffered considerable annual losses that are partially attributed to Colony Collapse Disorder (CCD). CCD is an unexplained phenomenon that correlates with elevated incidence of pathogens, including RNA viruses. Honey bees are eusocial insects that live in colonies of genetically related individuals that work in concert to gather and store nutrients. Their social organization provides numerous benefits, but also facilitates pathogen transmission between individuals. To investigate honey bee antiviral defense mechanisms, we developed an RNA virus infection model and discovered that administration of dsRNA, regardless of sequence, reduced virus infection. Our results suggest that dsRNA, a viral pathogen associated molecular pattern (PAMP), triggers an antiviral response that controls virus infection in honey bees.

## Introduction

Honey bee (*Apis mellifera*) pollination is a vital component of global food production (i.e., almonds, citrus, berries, alfalfa). Recent increased annual losses of honey bee colonies, partially attributed to Colony Collapse Disorder (CCD), have motivated correlative analyses of the parasitic (i.e., viruses, bacteria, fungi, mites) and environmental (i.e. chemical exposure, forage availability) threats to honey bee health [1-3]. Although the cause of CCD remains unknown, CCD-affected colonies have a greater number of pathogens, including RNA viruses, than healthy colonies [1-5]. Most honey bee viruses are non-enveloped, positive-sense single-stranded RNA (+ssRNA) viruses of the *Picornavirales* order. Virus infections may be asymptomatic, cause deformities, paralysis, or death in infected individuals (reviewed in 6). The genomes of numerous bee viruses including: acute bee paralysis virus (ABPV) [7], black queen cell virus (BQCV) [8], Israeli acute bee paralysis virus (IAPV) [9], Kashmir bee virus (KBV) [10], deformed wing virus (DWV) [11], Kakugo virus (KV) [12], sacbrood virus (SBV) [13], chronic bee paralysis virus (CBPV), and the Lake Sinai viruses [3,5,14] have been sequenced but infectious clones have not been developed. 

 Honey bees are eusocial insects that live in colonies of up to 50,000 individuals [7,15-19]. High population density within bee hives and behaviors such as mouth-to-mouth food exchange (trophallaxis) facilitate pathogen transmission [8,17,20,21]. Unlike solitary insects, bees benefit from social immune behaviors (i.e., grooming and behavioral fever) that may reduce colony pathogen burden (reviewed in 9,22,23). A critical number of bees are required to maintain colony temperature, rear brood (young bees), and carry out food gathering and storage. Thus an effective immune system is important at both the individual bee and entire colony level.

 Innate immune responses are the critical first line of host antiviral defense. Activation of these immune responses occurs via host recognition of non-self molecules or pathogen associated molecular patterns (PAMPs), including virally produced dsRNA. Seminal discoveries in diverse organisms including rice, fruit flies, and mice revealed that innate (non-specific) immune responses triggered by PAMPs are recognized by host pattern recognition receptors (PRRs) (reviewed in 3,10,24). In mammals, dsRNA is recognized by PRRs including dsRNA-dependent protein kinase (PKR), Retinoid-acid inducible gene I (RIG-I) [11,25,26], Melanoma differentiation-associated gene 5 (MDA-5) [12,27-32], and Toll-like receptor 3 (TLR3) [13,33,34]. Receptor binding of dsRNA triggers signal transduction cascades that inhibit translation and activate the interferon response, which results in transcriptional activation of hundreds of interferon-stimulated genes (ISG) involved in the antiviral state (reviewed in 35-38). 

 Recent studies indicate that in plants and invertebrates RNA interference (RNAi) plays a central role in antiviral immunity (reviewed in 39,40). RNAi is a sequence specific, post-transcriptional gene silencing mechanism that is triggered by dsRNA. Direct evidence of the antiviral role of RNAi in insects has predominantly come from studies in *Drosophila melanogaster*, *Aedes aegypti*, and *Anopholes gambiae* [15,41-49]. Similarly, studies in *Apis mellifera* (European or Western honey bee) [50,51] and *Apis cerana* (Asian or Eastern honey bee [52] implicate the role of RNAi-mediated antiviral immunity in honey bees. In addition to RNAi, the insect immune repertoire includes the Toll, Imd (for immune deficiency) and Jak/STAT (for Janus kinase and Signal Transducer and Activator of Transcription) innate immune response pathways (reviewed in 53,54). The importance of these pathways in insect antiviral defense is variable and specific to individual virus-host interactions [54-60]. 

 Bioinformatic analysis identified honey bee immune pathway members and determined that honey bees encode fewer immune genes than *Drosophila melanogaster, Aedes aegypti*, and *Anopholes gambiae* [22,25,61]. Previous investigations of honey bee immune responses have focused on immune responses to bacteria [22,62], fungi [63-69], a trypansomatid [69], and mites [70-72]. Investigation of honey bee antiviral responses has been hindered by lack of infectious honey bee virus clones, although some studies have been performed using semi-purified viruses and monitoring symptoms (reviewed in 6), colony size and weight [73], select gene expression [74], or antimicrobial peptide production in the context of either bacterial infection [75,76] or mite infestation [72]. RNAi-mediated antiviral immunity has been implicated [50-52,73], however honey bee antiviral defense mechanism(s) remain largely uncharacterized. 

 In this study, we report the development of an experimental model of honey bee virus infection, Sindbis virus expressing enhanced green fluorescent protein (SINV-GFP) [15,77]. We examined virus infection in the presence and absence of virus-sequence specific and non-specific dsRNAs. Based on evidence from honey bees [50-52] other insects (i.e., fruit flies and mosquitoes) [50,73], we expected that sequence specific RNAi would be the major honey bee antiviral defense mechanism. Instead, we determined that co-injection of non-sequence-specific dsRNA reduced virus copy number. This reduction suggests that dsRNA, a viral PAMP, triggers additional immune responses in honey bees. Importantly, a non-specific dsRNA-triggered antiviral response has not been observed in other adult insects. Transcriptional level response to virus and dsRNA, evaluated using honey bee gene expression microarrays, indicated that the majority of genes with previously characterized roles in insect immunity were not appreciably regulated by virus or dsRNA, suggesting that dsRNA-mediated antiviral defense in honey bees may involve unique genes and signal transduction cascades, alter or suppress known immune pathways, or may be predominantly post-transcriptionally regulated. 

## Results

### Honey Bee Virus Infection Model

**Figure 1:**
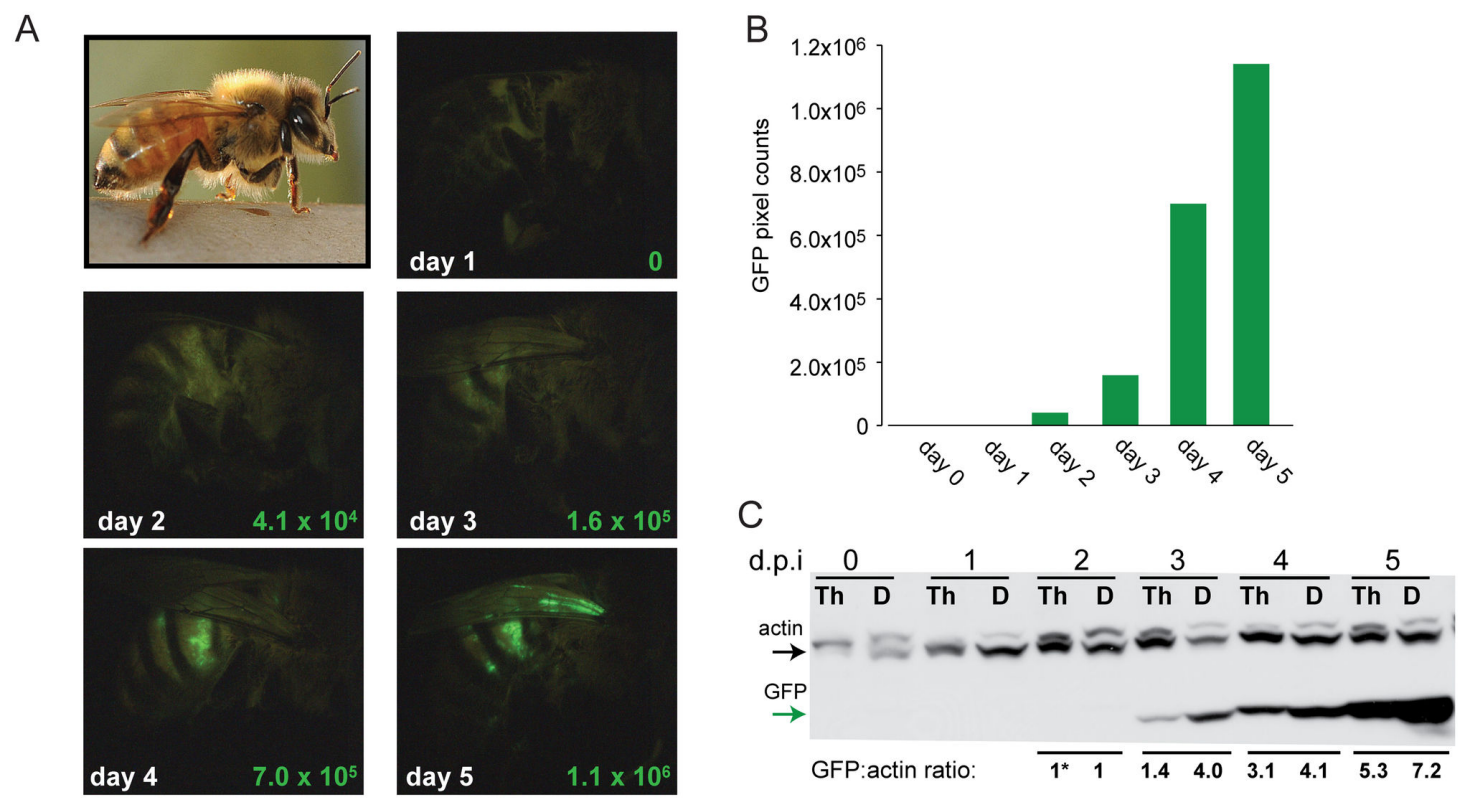
Honey bees are productively infected with a model virus, SINV-GFP. (A) Honey bees were infected with SINV-GFP and fluorescence microscopy revealed increased amounts of GFP each day post-injection (d.p.i.) (GFP pixel counts quantified using ImageJ in lower right corner of each image) and represented graphically in (B). (C) SINV-GFP was detected in honey bee protein lysates from the injection site, thorax (Th), and distal sites (D; head and abdomen) each d.p.i. by Western blot analysis using α-GFP (green lower arrow) and a-actin (top arrow) antibodies. Western blot bands were quantified using ImageJ and the GFP:actin ratio was normalized to 1* at day 2 p.i..

The majority of honey bee viruses are positive sense single-stranded RNA (+ssRNA) viruses that replicate via a dsRNA intermediate. Although the genomes of numerous honey bee viruses have been sequenced, there are no infectious clones available [78]. Since many insect viruses have broad host range we tested the ability of Sindbis virus (SINV) to infect honey bees. To facilitate monitoring virus infection we employed a recombinant virus, SINV-GFP, that expresses green fluorescent protein (GFP) [15,77]. Bees were inoculated with SINV-GFP via intra-thoracic injection and monitored daily. GFP fluorescence increased over time and spread throughout the bee (Figure 1 and S1). SINV-GFP is most readily visualized in the abdomen due to the relatively thin exoskeleton in this region (Figure 1A-B). The accumulation of virus in protein lysates from both the injection site (thorax) and distal regions (head, abdomen) demonstrated that SINV-GFP infection was systemic (Figure 1C and S1) and the ability to infect naïve bees with lysates from SINV-GFP infected bees demonstrated its infectivity (Figure S1). Relative abundance of SINV-GFP in individual, dissected bees was assessed by RT-qPCR and demonstrated productive infection and virus spread from the injection site (thorax) to distal regions (head and abdomen) (Figure S2). Together these data established that SINV-GFP productively infects honey bees and thus may function as a model of virus infection. Infection with a model virus has several advantages including the ability to precisely control the titer of virus inoculum and track the virus during infection. Intra-thoracic injection, as opposed to an oral infection route, ensured that each bee received the same infectious dose, and mimics mite (*Varroa destructor*) vectored honey bee virus infection [79-82]. The use of a model virus also ensured that honey bees, obtained from managed colonies housed outside of the laboratory, were not previously infected with SINV. This is particularly important to honey bee host-pathogen interaction research, since bees are readily infected with pathogens and their pathogen status has been show to vary on a weekly basis [3]; therefore, the pathogen status of individual bees at the onset of an experiment is unknown. Use of a model virus inoculum, rather than a semi-purified bee virus inoculum, ensures that all experimental subjects have the same initial dose at the onset of an experiment. While there are advantages and disadvantages to model and natural virus infection systems, both are required to furthering our understanding of honey bee antiviral immune responses. 

### dsRNA-mediated Antiviral Immunity in Honey Bees

**Figure 2:**
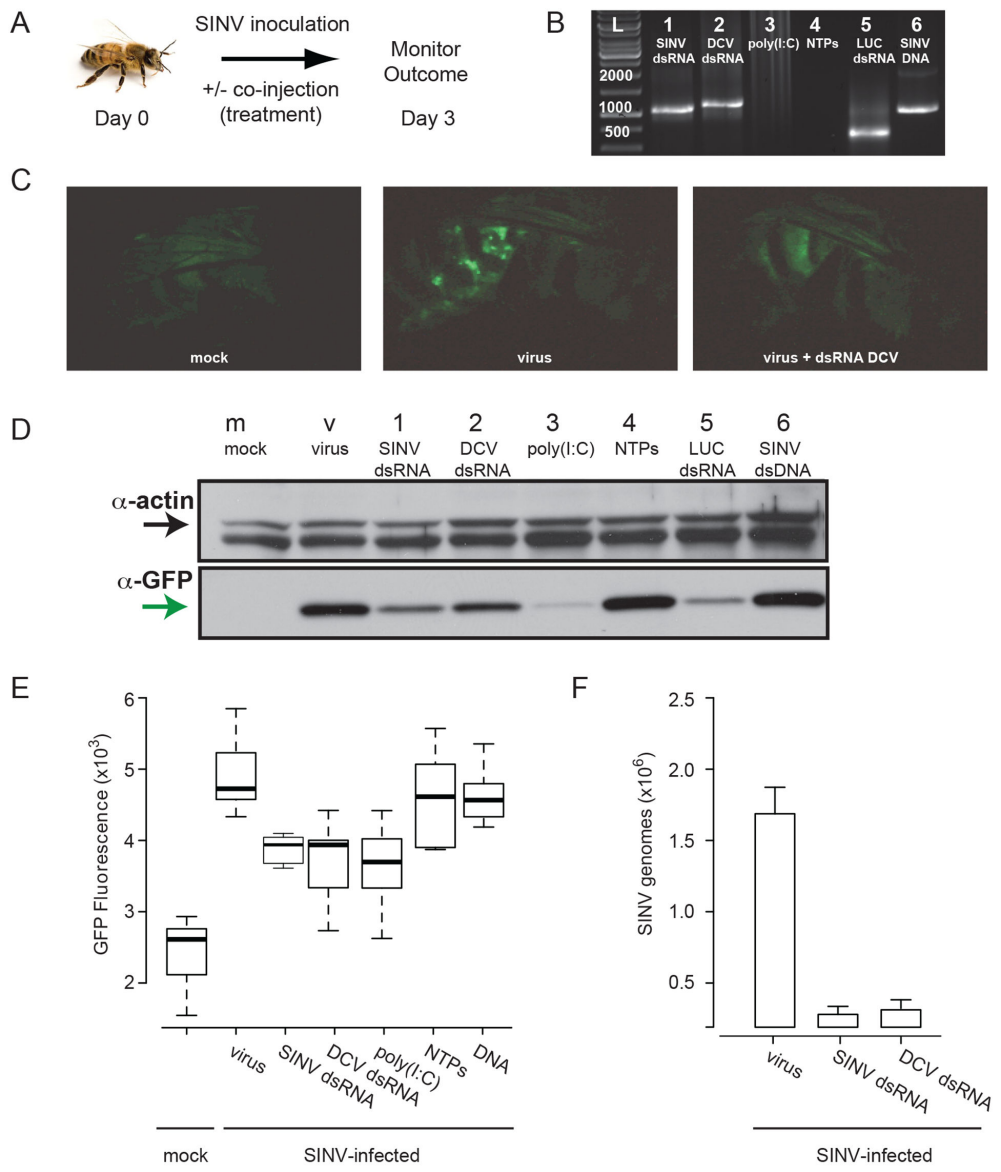
dsRNA-mediated reduction of virus in honey bees. (A) Schematic of experiment. Honey bees were inoculated with SINV-GFP or co-injected with (B) nucleic acid treatments including: virus-specific dsRNA (SINV dsRNA); non-specific dsRNA corresponding to Drosophila C virus (DCV) sequence (DCV dsRNA); poly(I:C); NTPs; non-specific dsRNA corresponding to luciferase sequence (LUC dsRNA); or SINV dsDNA visualized on a 1% agarose gel. (C) Fluorescence microscope images of a mock-infected bee (left panel), a SINV-GFP infected bee (middle panel), or a bee infected with SINV-GFP in the presence of non-specific dsRNA DCV. (D) Western blot analysis using α-GFP and α-actin antibodies demonstrated reduced SINV-GFP in bee protein lysates pooled from ten bees treated with dsRNA (lanes 1, 2, and 5) and poly(I:C) (lane 3), but not in bees treated with NTPs (lane 4) or dsDNA (lane 6) compared to bees inoculated with virus alone (virus). Mock-infected (mock) bees injected with buffer had no detectable GFP. (E) Boxplot of GFP fluorescence (510 nm, relative arbitrary units) in individual honey bee lysates (10 bees per condition). A Welch two-sample t-test was performed to compare honey bee lysates from each treatment group to virus-infected bees and determined statistically significant differences between bees co-inoculated with virus-specific dsRNA SINV p=0.023, with non-specific dsRNA DCV p=0.020, and with poly(I:C) p=0.0038, whereas co-injection of NTPs p=0.41 or dsDNA p=0.36 were not significantly different from virus alone (virus). Bees injected with virus only (virus) exhibited greater fluorescence compared to mock-infected controls (m); p=0.00001. (F) Relative abundance of SINV-GFP in individual bees (5 per condition) from different treatment groups was assessed by RT-qPCR. The bees treated with virus only had the highest SINV copy number 3 days p.i. (1.65x10^6^ copies + 4,100 copies per 500 ng RNA, ~1.65x10^8^ per bee), as compared to those in which either virus-specific SINV dsRNA (1.66x10^5^ +1,050) or non-specific DCV dsRNA (2.32x10^5^ + 1,070) was co-injected with virus.

RNA viruses generate long dsRNA molecules during their replication cycle, which trigger mammalian innate immune responses and serve as substrates for virus-specific RNAi-mediated antiviral immunity in plants and insects. To investigate the role of dsRNA in honey bee antiviral immunity, bees were injected with virus (SINV-GFP) alone or co-injected with multiple species and lengths of dsRNA including virus-specific dsRNA (SINV dsRNA, 928 bp), non-specific dsRNA corresponding to Drosophila C virus sequence (DCV dsRNA, 1017 bp and 448 bp), luciferase sequence (LUC dsRNA, 355 bp) and polyinosinic-polycytidylic acid (poly(I:C)), a synthetic mimic of dsRNA, the building blocks of dsRNA nucleotide-triphosphates (NTPs), or dsDNA (SINV dsDNA 928 bp) (Figure 2A-B). Three-days post-injection virus abundance was assessed by microscopy, Western-blot analysis, and qPCR. In *Drosophila*, sequence-specific dsRNA triggers an RNAi-mediated mechanism of antiviral immunity, whereas non-specific dsRNA has no effect [15]. Surprisingly, co-injection of non-specific dsRNA dramatically reduced virus infection in honey bees (Figure 2). Fluorescence imaging provided qualitative evidence of this reduction (Figure 2C) and quantitative data was obtained at the protein (or viral capsid level) from Western blot and fluorimetry analyses of bee lysates, and at the genomic level by quantitative PCR (qPCR) (Figure 2 D-F and Figure S3). Analysis of virally produced GFP-protein in pooled lysates (representing 10 bees each) demonstrated that co-injection of multiple species and lengths of dsRNA (SINV dsRNA, DCV dsRNA, LUC dsRNA, and poly(I:C)) decreased Sindbis virus production as compared to bees injected with virus only (Figure 2C). In order to rule out the possibility that dsRNA (a long poly-anion) was simply interfering with viral infection, dsDNA of the same length and sequence (SINV dsDNA) was co-injected with virus and no reduction in infection was observed (Figure 2 C-D, Figure S3). In addition, co-injection of nucleotide-triphosphates (NTPs), the building blocks of RNA present in excess in dsRNA production reactions, did not reduce SINV-GFP infection (Figure 2C-D and Figure S3). Fluorescence measurement of individual bee lysates validated differences between dsRNA-treated bees as compared to those co-injected with NTPs or dsDNA (Figure 2E). Likewise, Western blot analysis of individual bees supported data from pooled samples demonstrating dsRNA-mediated reduction in virus load, but also identified experimental outliers (Figure S3). These individuals were not further analyzed in this study and may be attributed to genetic differences between individual members of this out-bred honey bee colony. The average GFP to actin ratio in Western blots of individual bee lysates was 3.0 for virus-infected bees, whereas the ratio in bees that were co-injected with virus-specific dsRNA (SINV dsRNA), non-specific dsRNA (DCV dsRNA), or poly(I:C) was reduced to 1.2, 0.9, and 1.2 respectively (Figure S3). In contrast, the GFP to actin ratio was not as dramatically reduced in bees co-injected with NTPs, 2.3, or SINV dsDNA 2.4 (Figure S3). Overall the majority of bees that were individually assayed by Western-blot exhibited strong (> 50% less virus-GFP) dsRNA-mediated reduction in virus production. Specifically, 58% of bees co-injected with SINV dsRNA (n=66) and 69% of bees co-injected with DCV dsRNA (n=88) exhibited more than 50% reduction in virus-GFP. In contrast, only 3% of bees co-injected with NTPs (n=37) and 10% of bees co-injected with SINV dsDNA (n=20) had low levels of virus-GFP, which were likely a consequence of ineffective injections since these percentages were similar to the percentage of bees that were not infected after intra-thoracic infection of virus (8%, n=87). Western blot analysis was used to routinely assess dsRNA-mediated knock down of virus in bees and quantitative PCR (qPCR) was used to determine the relative abundance of SINV-GFP genome copies in a subset of differentially treated individuals (Figure 2F). Bees injected with only virus and buffer had the highest SINV genome copy number (1.65x10^6^ + 4,100 copies) 3 days p.i. relative to those treated with either virus specific dsRNA (SINV dsRNA) or non-specific-dsRNA (DCV dsRNA) in conjunction with virus, 1.66x10^5^ +1,050 and 2.32x10^5^ + 1,070 average genome copies, respectively. We conclude that co-injection of non-specific dsRNA effectively limited Sindbis virus infection in honey bees. 

###  Transcriptional level evaluation of virus and dsRNA induced immune responses

We hypothesized that some of the honey bee genes involved in antiviral defense would be transcriptionally regulated and that a subset of these genes would also be regulated in response to dsRNA, a viral PAMP. In order to both examine the role of previously characterized insect immune genes and potentially identify additional honey bee host factors responsible for antiviral defense, we utilized custom oligonucleotide microarrays to evaluate the transcriptional profiles of virus-infected or dsRNA-treated bees [27,31-33,83].

 Long-term laboratory rearing of honey bees is not feasible, therefore we obtained frames of brood from colonies outside the laboratory. To minimize genetic variability between samples, all microarrayed bees were obtained from a single brood comb from a naturally-mated queen and were therefore age-matched half-sisters. Bees that co-emerged in the lab were grouped into samples of 10 and then intrathoracically injected with SINV-GFP virus, dsRNA (dsRNA SINV 928 bp; 1 μg), or buffer (mock-infected). After 3 days, honey bee samples were individually analyzed by Western-blot, fluorimetry, and qPCR as described above. In addition, since bees are readily infected with pathogens we utilized the arthropod pathogen microarray (APM), which is capable of detecting all common honey bee pathogens (at levels greater than 10,000 genome copies) and over 200 additional arthropod pathogens [3], to screen for pre-existing conditions prior to transcriptome analysis. APM profiling ensured that the predominant infection and prevailing immune response in the bees analyzed by microarray analysis was due to the experimentally introduced SINV-GFP or dsRNA and not a consequence of pre-existing infections (Table S1). All microarrayed samples were also tested for deformed wing virus (DWV), a common honey bee pathogen detected in APM analysis of two RNA-treated bees (Table S1). DWV qPCR determined that all the samples used for transcriptome analysis had DWV levels less than 10^4^ copies of virus per 500 ng RNA (< 10^5^ copies per bee) (Table S1), whereas SINV-GFP levels were approximately 10^6^ copies per 500 ng RNA (~ 10^8^ copies per bee) (Figure 2, Table S1). Levels of common bee viruses are typically more than 10^6^ copies per 100 ng RNA (> 5x10^8^ copies per bee) [3,84-86]. In general, bees that emerged in laboratory conditions had less infections than older bees obtained from colonies housed outside of the laboratory [3] (Table S1, positive controls). 

 We identified five representative bees from each group (mock-infected, SINV-GFP infected, or dsRNA-treated) that were relatively free of honey bee pathogens and analyzed gene expression at the transcriptional level using honey bee oligonucleotide microarrays. Individual bee transcriptomes at three-days post-injection were examined since at this time-point the virus was actively replicating and spreading at levels detectable by Western blot (Figure 1D), no secondary ill-effects of laboratory housing were observed, and this was the time-point used to assess dsRNA-mediated reduction of virus. Also, we reasoned that the wound-response due to injections (although controlled by mock-infection) would be reduced by three days post-injection, thus facilitating investigation of virus and dsRNA-triggered responses. 

**Figure 3:**
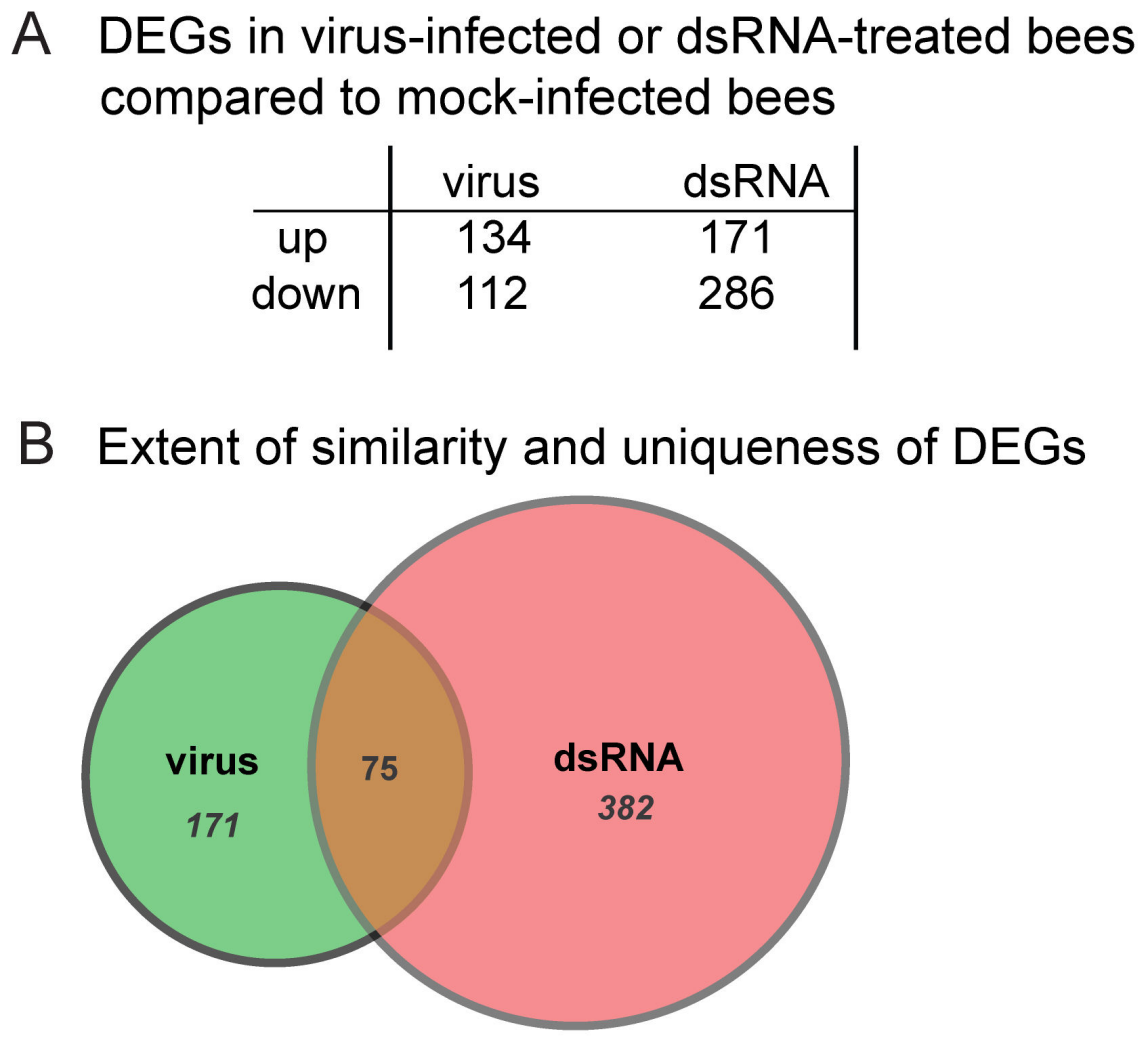
Differentially expressed honey bee genes. (A) Table of differentially expressed genes (DEGs) in virus-infected or dsRNA-treated bees as compared to mock-infected bees (adj. p-value < 0.05); the number of genes with increased (up) and decreased (down) expression are listed below each condition. (B) Venn diagram illustrating the degree of similarity and uniqueness of DEGs (adj. p-value < 0.5) between virus-infected (virus) and dsRNA-treated bees (dsRNA).

 Custom honey bee gene expression microarrays were utilized to identify genes that were differentially expressed in virus-infected and dsRNA-treated bees compared to mock-infected (buffer-injected) bees. The honey bee oligonucleotide microarray has features representing 9,867 unique genes [25], 2,729 EST probes, 22 probes specific for honey bee pathogens, and control spots including 11 probes for green fluorescent protein (GFP) (Table S2) [27,29-32]. Experiments were designed to meet Minimum Information About a Microarray Experiment (MIAME) standards and the protocols utilized for cDNA synthesis, labeling, hybridization, analysis (R-code for LIMMA) and the raw microarray data obtained in this study were deposited at ArrayExpress [www.ebi.ac.uk/arrayexpress (accession no. E-MEXP-3608)]. To facilitate gene expression comparisons we utilized a reference-design strategy in which each Cy5-labeled cDNA experimental sample was hybridized with a standardized Cy3-labeled reference honey bee cDNA sample. Microarrays were scanned and global analysis of the differentially expressed genes (DEGs) was performed using Bioconductor’s Linear Models for Microarray Analysis (LIMMA) [35] implemented in the R/Bioconductor platform [87] and data from duplicate array features was condensed into a single value for statistical analysis [16,19]. The data from the five individual arrays in each treatment group were analyzed in a pairwise comparison to five individual arrays from the mock-infected (buffer-injected) group using a contrast matrix. The data were fit to a linear model using EBAYES and false-discovery rate FDR p-value <0.05; and an adjusted p-value, which accounts for multiple pairwise comparisons, less than 0.05 (adj. p-value < 0.05) was selected to define significant differences between each experimental condition as compared to mock-infection. Using these criteria, 134 genes were induced and 112 genes exhibited decreased expression levels in response to virus-infection; dsRNA-treatment resulted in the induction of 171 genes and reduced expression of 286 genes (Figure 3A). Seventy-five DEGs were shared between virus-infected bees and dsRNA-treated bees (Figure 3B, Tables S3-S5). 

**Figure 4:**
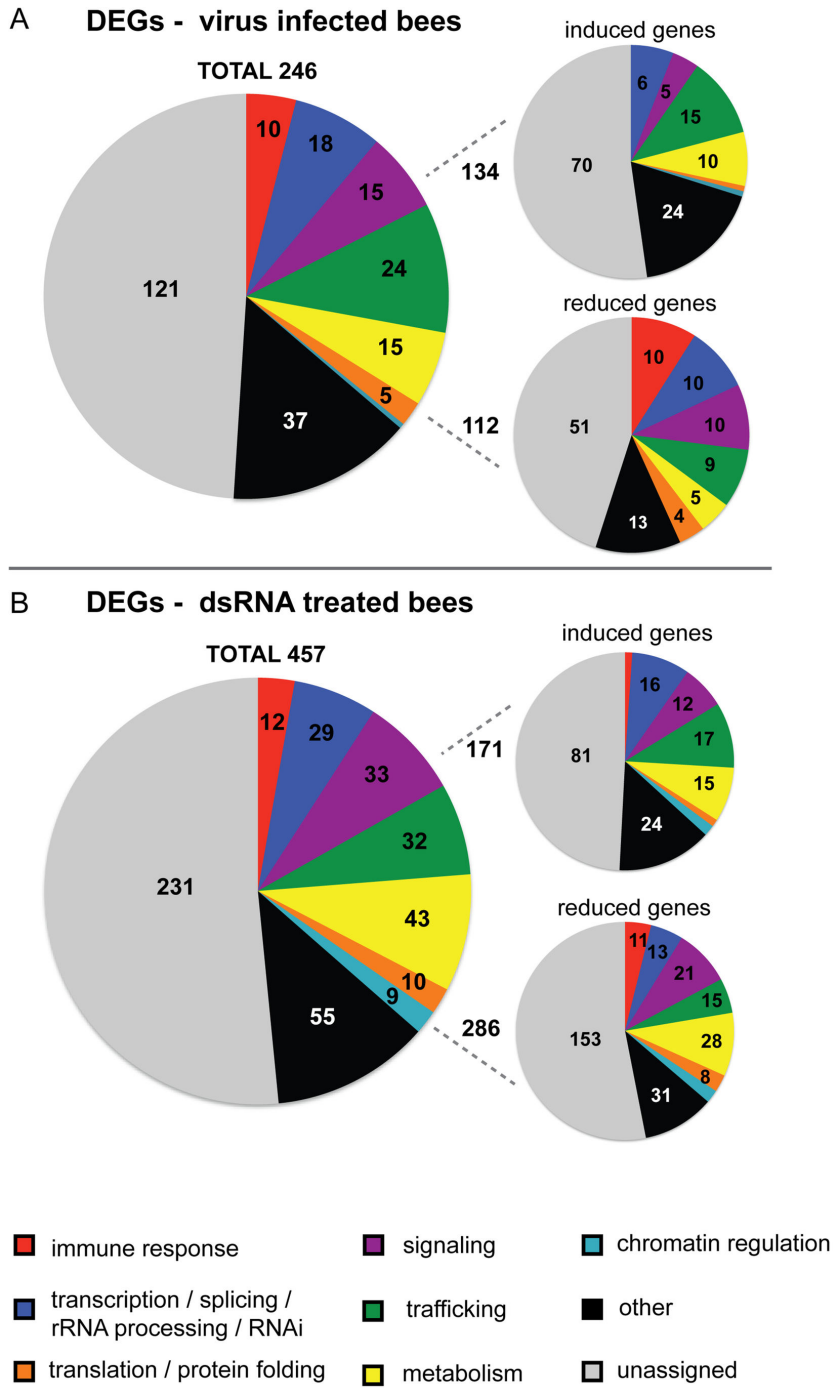
Gene Ontology analysis demonstrated reduced expression of immune genes and perturbation of cell-signaling, trafficking, and metabolism. Differentially expressed genes (DEGs, adj. p-value < 0.05) in (A) virus-infected or (B) dsRNA-treated honey bees as compared to mock-infected bees were assigned gene ontology terms for biological process, molecular function, and cellular compartment (see Tables S3-4). Biological process assignments were further categorized into seven functional groups: immune response (red); transcription, splicing, rRNA processing and RNAi (blue); signaling (purple); trafficking (green); metabolism (yellow); translation and protein folding (orange); and chromatin regulation (teal). A small fraction of the DEGs (10-15%) belonged to other biological process gene ontologies (black) and a large fraction was not assigned (gray). Each pie chart represents 100% of the DEGs and gene numbers are reported in each section. *Each oligonucleotide pair was analyzed as a single gene and since multiple microarray probes corresponded to apidaecin variants the majority of annotated immune effectors in this Figure (7 of 10 in virus-infected bees and 8 of 12 in dsRNA) correspond with apidaecin 1.

 To globally evaluate the putative function of transcriptionally regulated genes in response to virus-infection and dsRNA-treatment, Gene Ontology (GO) annotations were assigned to all *Apis mellifera* microarray probes with *Drosophila melanogaster* orthologs in terms of their associated biological processes, molecular functions, and cellular components (Tables S3-S4) [27,32,88]. Biological process assignments were further categorized into seven functional groups: immune response, including honey bee AMPs; transcription; splicing; rRNA processing and RNAi; signaling; trafficking; metabolism; translation and protein folding; and chromatin regulation (Figure 4 and Tables S3-S5). This analysis indicated reduced expression of several immune genes and perturbation of cell signaling, trafficking and metabolism in virus-infected or dsRNA-treated honey bees (Figure 4). Over-representation analysis, for pathways with more DEGs that would be expected by chance, identified pathways in virus-infected bees (i.e., the eicosanoid signaling pathway, p-value=0.009) and dsRNA-treated bees (i.e., oxidative phosphorylation, p=0.00005) (Table S6). 

**Figure 5:**
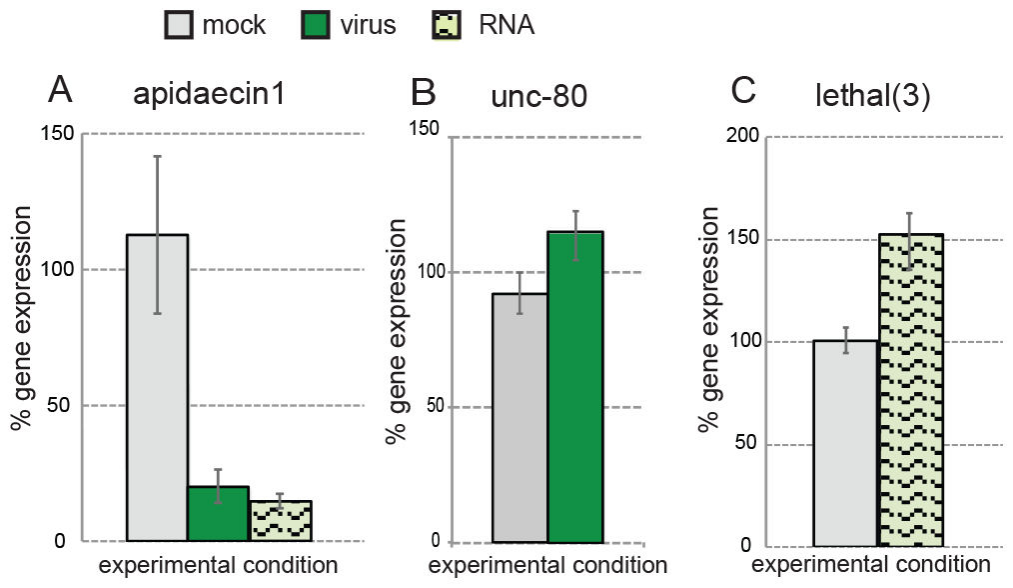
Quantitative PCR validation of a subset of DEGs. Quantitative PCR (qPCR) was used to validate honey bee gene expression microarray results. (A) apidaecin 1 expression in virus-infected bees was 20% + 6 of mock-infected controls (112% + 30%) and dsRNA-treatment also reduced expression to 15% + 3% of mock-infected controls; t-test p=0.01 and p=0.01 respectively. (B) unc-80 expression in virus-infected bees (126% + 9%) was higher than mock-infected controls (101% + 4%) (p=0.046). (C) lethal (3) expression increased in response to dsRNA-treatment to 156% + 14 relative to its expression in mock-infected bees 101% + 6% (p=0.03. Standard SYBR qPCR was performed for apidaecin 1 gene expression, whereas Taqman probe and primer sets were employed for other assayed genes. qPCR reactions were performed as technical triplicates of five individual bee cDNAs (biological replicates) per condition; the expression of each gene-of-interest (GOI) was compared to the housekeeping gene (Rpl8) and then the expression level of each GOI in five individual bees per experimental condition was calculated as a percentage relative to its expression in individual mock-infected bees using the ΔΔC(t) formula (y-axis), error bars represent +/- standard error of the mean (SEM), and statistical significance was assessed using Student’s t-tests.

 The most significant differentially expressed genes were the antimicrobial peptide (AMP) encoding genes *apidaecin* and *hymenoptaecin*, which exhibited decreased expression in both virus-infected and dsRNA-treated bees (Table 1). Reduction of *Apidaecin 1* was verified by qPCR analysis; expression in virus-infected bees was 20% + 6% that of mock-infected controls and was similarly reduced to 14.5% + 3% in dsRNA-treated bees (Figure 5A). As expected, the microarray probes corresponding to GFP were identified as the most induced genes in the virus (SINV-GFP) infected group, accounting for 11 of the top 15 induced probes identified (Table 2). This result, which was confirmed by SINV-GFP qPCR analysis, also validated our microarray analysis methods. Additional genes that were identified by microarray analysis and verified by qPCR include increased expression of *unc-80* in virus-infected bees and *lethal*(3) in dsRNA-treated bees (Figure 5). 

**Table 1:** Identification of the honey bee transcripts with the greatest decrease in expression in response to virus infection or dsRNA-treatment.

**Genes with reduced expression in virus-infected honey bees (FC < 2)**			
**ArrayID**	***Apis m.* (NCBI)**	**FC**	**Name /Function**
AM00353	XM_003249457.1	-5.5	apidaecin (Apid73)
AM00358	NM_001011613.1	-3.8	apidaecin 1 (Apid1)
AM00354	NM_001011642.1	-3.4	apidaecin type 22
AM00351	NM_001011642.1	-2.8	apidaecin type 22
AM10109	NM_001011615.1	-2.6	hymenoptaecin
AM00360	NM_001011642.1	-2.5	apidaecin type 22
AM00355	NM_001011642.1	-2.4	apidaecin type 22
AM00361	NM_001011613.1	-2.2	apidaecin 1
AM00359	NM_001011613.1	-2.1	apidaecin 1
AM08876	XM_001121619.2	-2.1	diphosphomevalonate decarboxylase-like
AM10888	NM_001011617.1	-2.0	abaecin
**Genes with reduced expression in dsRNA-treated honey bees (FC < 2)**			
**ArrayID**	***Apis m.* (NCBI)**	**FC**	**Name /Function**
AM00353	XM_003249457.1	-3.0	apidaecin
AM00358	NM_001011613.1	-2.8	apidaecin 1
AM00360	NM_001011642.1	-2.6	apidaecin type 22
AM02920	XM_397549.4	-2.5	DENN domain-containing protein 1A-like v2
AM00354	NM_001011642.1	-2.5	apidaecin type 22
AM00351	NM_001011642.1	-2.4	apidaecin type 22
AM00359	NM_001011613.1	-2.3	apidaecin 1
AM06107	XM_625146.3	-2.1	hydroxyacid oxidase 1
AM12438	XM_392810.3	-2.1	fem-1 homolog
AM00356	XM_003249457.1	-2.0	apidaecin

Table abbreviations are as follows: microarray feature identification number = Array ID; honey bee NCBI reference number = *Apis m.* (NCBI); fold-change = FC; and Name/Function.

**Table 2:** Identification of the honey bee transcripts with the greatest increase in expression in response to virus infection or dsRNA-treatment.

**Genes induced in virus-infected honey bees (FC > 2)**			
**ArrayID**	***Apis m.* (NCBI)**	**FC**	**Name /Function**
control		4.2	GFP tagged virus
AM00006	NM_001014991.1	4.0	DSCAM / IR
AM10238	NM_001172378.1	3.5	Cpap3-b / chitin binding
AM06483	XM_001122676.2	3.0	unc-80 / endocytosis
AM03972	XM_624537	2.7	short-chain dehydrogenase reductase (SDR) family member 11-like
AM05204	XM_392552	2.5	fruitless / reproduction, zinc finger DNA binding
AM05793	XM_624451.3	2.5	glucosinolate sulphatase
AM00965	XR_120004.1	2.1	NA
AM04232	XM_392262.3	2.1	SET and MYND domain protein 4-like
**Genes induced in virus-infected honey bees (FC > 1.8)**			
**ArrayID**	***Apis m.* (NCBI)**	**FC**	**Name /Function**
AM02966	XM_397160.3	2.6	lethal (3) /rRNA biogenesis
AM04958	XM_394432.4	2.4	nemo / Serine/threonine-protein kinase
AM05006	XM_623595.2	2.0	coiled-coil domain-containing protein 12
AM09047	XM_392018.4	1.9	NAD^+^ kinase activity
AM03325	XM_395638.4	1.8	Plenty of SH3s (POSH) / SH3 domain-containing RING finger protein 3
AM07355	XM_003250568.1	1.8	hypothetical protein

Table abbreviations are as follows: microarray feature identification number = Array ID; honey bee NCBI reference number = *Apis m.* (NCBI); fold-change = FC; and Name/Function.

 We examined the expression of genes involved in insect immunity, as well as additional genes identified in transcriptional studies of virus-infected fruit flies and mosquitos, in virus-infected and dsRNA-treated honey bees (Table S7) [22]. Specifically, our candidate gene analysis included genes in the Toll, Imd, and JAK-STAT signaling cascades, as well as those involved in dsRNA uptake and RNAi-mediated antiviral immunity in fruit flies and mosquitos [15,39,41-48]. The majority of these genes (95%), even those that are transcriptionally regulated in other insects, were not differentially expressed in honey bees three days post SINV-GFP infection (Table S7). This analysis suggests that at three days post-infection only *clathrin heavy chain*, which is involved in endocytosis, and *eater-like* (*nimrod C1*), which encodes an EGF-motif containing protein involved in phagocytosis, were significantly activated in virus-infected honey bees (Table S7). Together the transcriptional data presented herein suggest that antiviral defense in honey bees may involve unique genes, alter or suppress known immune pathways, or may be predominantly post-transcriptionally regulated.

## Discussion

Honey bees are agriculturally important eusocial insects that are readily infected with ssRNA viruses. Virus infections may be rapidly cleared or result in death of individual bees and entire colonies [3,6,89,90]. Although the outcome of infection depends critically on the honey bee antiviral immune response, the mechanisms involved in mitigating honey bee viral infections are not well-characterized. In this study we established a model of honey bee virus infection and determined that co-administration of dsRNA, regardless of sequence specificity, reduced virus infection. Our results suggest that that honey bees activate antiviral immune pathway(s) in response to non-specific dsRNA. Understanding these mechanisms is critical to assessing the role of viruses and/or immune deficiencies in colony losses and may lead to the development of strategies that enhance honey bee survival. 

 There are several challenges to investigating honey bee antiviral responses, including genetic heterogeneity within and between colonies, the prevalence of pre-existing infections, and the lack of infectious honey bee virus clones. To address these challenges we used age-matched half-sisters in experiments, screened for pathogens prior to gene expression analysis, and established SINV-GFP as a model for honey bee virus infection. Sindbis virus is a +ssRNA virus that has been used to investigate antiviral immunity in fruit flies and mosquitos. Importantly, SINV does not encode a suppressor of RNAi [45,91,92], so although it is in a different family of +ssRNA viruses (*i.e. Togaviridae*) than honey bee viruses, it is a useful model for investigation of dsRNA triggered immune mechanisms. Furthermore, use of this model virus facilitated monitoring infections using the GFP-tag, ensured no prior infection, and reduced the effects of virus-induced immune repression often observed in co-evolved virus-host pairs. 

 Previous research in fruit flies and mosquitos has shown that RNAi is the primary mechanism of antiviral defense, while the role of other immune pathways in mitigating virus infection varies with the specific virus-host pair. The honey bee genome encodes the RNAi machinery and orthologs of the Toll, Imd, JAK-STAT, and JNK immune pathways [22,25]. RNAi-mediated gene knock-down of endogenous genes has been utilized as a reverse genetics tool for examining gene function [18,93-98], although off target effects have also been reported [99]. RNAi has also been implicated in antiviral defense, although the molecular details require further investigation [50-52,73]. We investigated the role of dsRNA in honey bee antiviral immunity and determined that co-injection of both virus-specific and nonspecific dsRNA reduced virus production. This result was surprising in context of studies demonstrating virus-specific dsRNA mediated reduction of virus copy number and reduced honey bee lethality (*i.e.,* [50-52,73]). However, careful examination of the experimental details revealed several differences between the work presented herein, which examined the effect of co-injection of a single dsRNA dose (1 μg / bee) on the replication of a model virus, and other *Apis mellifera* studies, which examined treatment of larva and adults with dsRNA prior to infection by feeding [50-52], higher doses of dsRNA (6 μg / bee) [50], different doses of semi-purified viruses [50,51], various evaluation methods at different time-points post virus-inoculation [73], and one study that examined *Apis cerana* (Eastern honey bee) larva that were fed dsRNA prior to and during virus inoculation (5 μg dsRNA / larva) [52],; the experimental details may account for the differences observed. Clearly, additional studies are needed to more fully understand dsRNA-mediated immune responses in honey bees, which likely involve both virus-specific RNAi-mediated and non-specific dsRNA mediated mechanisms. 

 Interestingly, the dsRNA-mediated response we observed in honey bees also differed from that observed in fruit-flies infected with the same model virus, Sindbis-GFP [15]. In fruit flies, co-injection of virus-specific dsRNA triggers an RNAi-mediated antiviral response, whereas co-injection of non-specific dsRNA did not reduce virus titers [15]. In contrast, the general non-sequence-specific immune response to dsRNA that we observed in honey bees may be analogous to mammalian innate immunity. In mammals, several PRRs recognize dsRNA as a viral PAMP and signal innate antiviral responses, but to our knowledge the work described herein is the first report of non-specific dsRNA-mediated reduction of virus titer in adult insects. Interestingly, non-specific dsRNA-triggered antiviral immunity has also been reported in sand-fly cells [100], Chinese oak silk moth pupae [101], and shrimp (reviewed in 102], [13,103-107), and dsRNA-triggered transcriptional responses in *Bombyx mori* larva suggest that dsRNA is recognized as a PAMP [108]. However, the PRRs, genes, and pathways involved in these processes have not been characterized. Intriguingly, expression of antiviral immune response genes (i.e., vago and vir-1) in *Drosophila* [60,109] and mosquitos [110,111] and up-regulation of Dicer-2 in response to Dcr-dsRNA [112] and to GFP-dsRNA in *Bombyx mori* [108] indicates that Dicer-2 may also serve as dsRNA (PAMP) sensor, response element, and/or mediator of innate immune signaling. Cumulatively, these studies suggest that non-specific dsRNA mediated immune responses are important in arthropods; however future studies are needed to determine the molecular mechanisms and relative importance of this response in specific host - pathogen interactions. 

 Transcriptional profiling of virus and dsRNA-treated honey bees facilitated analysis of previously identified immune gene orthologs (Table S7; [22]) and putatively identified genes involved in virus and dsRNA triggered innate immunity (Tables 1-2). In our study, the majority of canonical insect immune pathway members did not appear to be transcriptionally regulated 3-days post injection with either virus or dsRNA (Table S7) [22]. Interestingly, antimicrobial peptide (AMP) encoding genes *apidaecin*, *hymenoptaecin*, and *abaecin*, exhibited significant decreased expression in virus-infected and dsRNA-injected bees relative to mock-infected bees (Table 1). These effector molecules are typically transcriptionally induced after insect immune challenge via Toll, Imd, or JNK signaling pathways [22]. Reduced AMP expression suggests that honey bee antiviral responses involve different effector molecules than those involved in antibacterial and antifungal immune responses. Intriguingly, parasitic mite infestation coupled with wing deformity and higher amounts of deformed wing virus (DWV) also reduced AMP and immune related enzyme expression in honey bees [72]. Likewise, AMP production was not induced by infection with acute bee paralysis virus (ABPV) [75] or in CrPV-infected fruit flies [59], whereas a positive correlation between hymenoptaecin expression and DWV titer was demonstrated in naturally infected honey bees [4]. The majority of genes that exhibited increased transcription in response to virus-infection or dsRNA-treatment were not canonical insect immune genes (Table 2 and Tables S3, S4 and S7). For example, virus-induced *A. mellifera unc-80*, whose function is unknown in *A. mellifera*, functions in vesicular trafficking and proper localization of NCA ion channels in *C. elegans*. Thus, we predict this protein functions similarly in honey bees. *Lethal* (3) expression increased in response to dsRNA and its function in honey bees is also unknown, but it may play a role in rRNA processing or splicing similar to its *Drosophila* and mammalian (NOP14 nucleolar protein) orthologs. It is noteworthy that several of the genes we identified by microarray analysis have multiple splice variants (i.e., *DSCAM* and *fruitless*). Interestingly, the induction and involvement of genes without previously characterized roles in immunity was also observed in *Drosophila* C virus (DCV)-infected fruit flies [60] and O'Nyong-Nyong virus (ONNV)-infected *A. gambiae* mosquitos [56]. Overall the transcriptional data presented herein suggest that antiviral defense in honey bees may involve unique genes and signal transduction cascades, alter or suppress known immune pathways, or may be predominantly post-transcriptionally regulated. Future studies involving more samples, additional time-points, and tissue-specific analyses are required to confirm the involvement of the genes identified herein, as well as further examine the potential role of canonical immune genes, in honey bee antiviral immune responses. 

 In summary, we established a virus infection model to investigate antiviral immunity in honey bees and determined that co-injection of dsRNA, regardless of sequence-specificity, mitigated virus infection. Our results suggest the involvement of an RNAi-independent immune mechanism. Future studies will focus on determining the mechanism and relative contribution of non-specific dsRNA-mediated antiviral immunity in honey bees. 

## Materials and Methods

### Honey bees

Honey bees are not well adapted to the laboratory setting, therefore for our experiments we used newly emerged honey bees (~24 hours old). Frames of emerging bees were obtained with permission from privately owned, managed colonies in San Francisco, CA. No additional permissions were required since the managed honey bee colonies were privately owned colonies and *Apis mellifera* is not an endangered or protected species. Young female worker bees (all half-sisters) that emerged in the laboratory were collected daily and housed in modified deli-containers (similar to those described by Evans et al. 2009 [113]) at room temperature for the duration of the experiment. Water and sugar were provided and replenished daily through the duration of the experiment. 

### Sindbis virus (SINV-GFP) production and injection into honey bees

Sindbis virus expressing enhanced GFP (SINV-GFP) was produced by transfecting *in vitro* transcribed RNA from the pTE3’2J with GFP inserted into the XbaI site into BHK cells [15,77]. SINV-GFP was titered on BHK cell monolayers via plaque assay. SINV-GFP (3,750 plaque forming units (PFUs)) was diluted in buffer (2 μl of 10 mM Tris, pH 7.5) and injected into the thorax using a Harbo syringe (from Honey Bee Insemination Service; www.honeybee.breeding.com/HarboAssembly.html) equipped with disposable borosilicate needles made from capillary tubes (0.8-1.10 x 100 m) using a micropipette puller (Sutter Instruments Model P-87). Honey bees were immobilized via incubation in a cold room (4°C) for 20 minutes and with insect pins and forceps during injection; after injection bees recovered at room temperature within 5 minutes. Honey bees were infected with 3,750 PFUs of SINV per bee. This virus dose is modest compared to *Drosophila* studies which typically utilize 250-2500 PFUs per fly; a newly emerged female worker honey bee (~150 mg) weighs ~ 200x more than an adult female fruit fly (0.8 mg)[15]. This dose allowed for a natural progression of infection over the course of the experiment and at 3-days post-injection (p.i.) virus abundance could be easily assessed by microscopy and Western-blot analysis. Virus was either injected alone or in conjunction with dsRNA (1 μg).

### dsRNA preparation

dsRNA was generated by *in vitro* transcription with T7 RNA polymerase [15,18]. In brief, T7 promoter containing dsDNA PCR-products (1-10 μg), amplified with the primers listed in Table S8, served as templates for T7 polymerase (NEB) transcription (100 μl reactions: rNTPs (each 7.5 mM final), RNase OUT (40 units) (Invitrogen), buffer (400 mM HEPES pH 7.5, 120 mM MgCl_2_, 10 mM Spermidine, 200 mM DTT); reactions were carried out at 37°C for 4-12 hours. DNA templates were removed by incubating with DNAse I (1 unit; Fermentas) for 15 minutes at 37°C. ssRNA products were ethanol precipitated, suspended in 200 μl DEPC-treated water, and annealed by heating the reaction to 100°C for 10 minutes and then slowly cooling to room temperature. dsRNA products were purified by phenol:chloroform extraction followed by ethanol precipitation; dsRNA for injection was suspended in 10 mM Tris pH 7.5. dsRNA quality and quantity were assessed by agarose gel electrophoresis and spectrophotometer (NanoDrop 2000c, ThermoScientific).

### Honey bee protein lysate preparation and analysis

Individual honey bees were homogenized in 400 μL buffer (10 mM Tris pH 7.5, 1X Complete Protease Inhibitor cocktail (Roche) in a 2 mL micro-centrifuge tube containing one sterile zinc-coated steel ball bearing (5 mm) using a TissueLyzer II (Retsch), for 4 minutes at 30 Hz. Lysates were clarified by spinning for 12 minutes at 12,000 x g. Fluorescent analysis of lysates was performed using a microplate reader (Safire, TECAN), and relative GFP abundance was measured by SDS-PAGE coupled with Western blot. For Western blot analysis, lysates were combined with Laemmli buffer (95°C for 3 min.), run on 4-12% acrylamide gels (Mini-PROTEAN TGX, BioRad), transferred to PVDF membrane (Immobilon-P), blocked with 5% milk in TBS buffer containing 0.1% Tween-20, incubated overnight with primary antibodies (anti-GFP sc-8334 or anti-actin sc-1616; Santa Cruz Biotech.), HRP-conjugated anti-rabbit secondary antibody (ECL, GE Healthcare), and developed with SuperSignal West Pico Chemiluminescent Substrate (Pierce). ImageJ was used to quantify the sum of the pixels within actin and GFP regions in order to calculate and compare SINV-GFP levels in samples [20,21]. 

### Honey bee RNA isolation and purification

TRizol reagent (Invitrogen) was added to individually homogenized bees and RNA was isolated according to manufacturer’s instructions and further purified using Qiagen RNeasy columns, including on-column DNase Treatment (Qiagen). RNA quality was assessed using an Agilent 2100 Bioanalyzer. 

### Quantitative PCR (qPCR)

cDNA synthesis reactions were performed with SuperScriptIII (Invitrogen) and random hexamer primers according to manufacturer’s instructions. qPCR was performed in triplicate wells using 2 μL of cDNA as template in 20 μl reactions composed of SensiFAST SYBR Mastermix (Bioline), MgCl_2_ (3 mM), and forward and reverse primers (600 nM each) on a CFX Connect Real Time instrument (BioRad). The qPCR thermo-profile consisted of a single pre-incubation 95°C (3 min), 40 cycles of 95°C (5 s), and 60°C (20 s) for SINV and *Apis m*. Rpl8 primer sets (Table S8). Target SINV qPCR amplicons were cloned into the pGEM-T (Promega) vector and sequence verified. Plasmid standards, containing from 10^9^ to 10^2^ copies per reaction, were used as qPCR templates to assess primer efficiency and generate the SINV-specific standard curve used to quantify the viral genome copy number. The linear standard equation generated by plotting the crossing point (Cp) versus the log_10_ of the initial plasmid copy number for the qSINV primer set was as follows: SINV Cp = -4.3 +40.3, R^2^ =0.977. The detection limit of this qPCR primer set was 10^3^ copies and specific qPCR amplicons had Cp values of < 26. Values obtained from the no RT control reactions were below the detection limit of the assay and subtracted from treatment group values before graphing. For DWV qPCR plasmid standards containing from 10^4^ to 10^7^ copies per reaction were used as qPCR templates to assess primer efficiency and generate the DWV-specific standard curve used to quantify the viral genome copy number. The linear standard equation was: DWV Cp = -3.3 +32.0, R^2^ =0.996. The detection limit of this qPCR primer set was 10^4^ copies and specific qPCR amplicons had Cp values of < 25. An estimate of the number of viral genomes per bee can be obtained by multiplying the reported qPCR copy number values by 100. This estimate is based on the following: typical RNA yield was approximately 50 μg per bee, and each qPCR reaction was performed on cDNA generated from 500 ng RNA, therefore each well represents 1/100^th^ of an individual bee. qPCR using a host primer set, *Apis m*. Rpl8, was also performed on pooled cDNA templates from each treatment group; the Rpl8 primer efficiency was evaluated using a cDNA dilution series (Cp = -3.5 + 17, R^2^ =0.996). Lastly, no-RT control reactions using pooled RNA as the template, and negative control reactions using water in place of template were performed in triplicate on each plate. Melt point analysis and 2% agarose gel electrophoresis ensured qPCR specificity. TaqMan® Fast Advanced Master Mix and Taqman® primer and probe sets were used for following *Apis mellifera* genes (target region; qPCR efficiency test): *unc-80* (100-250; Cp = -3.2 +25.5, R^2^ =0.99), *lethal*(*3*) (600-800; Cp = -3.8 +25.5, R^2^ =0.99) (Applied Biosystems). The ∆∆C(t) method was used to calculate the expression of the gene-of-interest (GOI) in individual bees. In brief, ∆C(t) for each sample was calculated by subtracting the Rpl8 C(t) from the GOI C(t). The ∆∆C(t) was calculated by subtracting mock-infected ∆C(t) values from the ∆C(t) values associated with each treatment group (5 individual bee samples per condition). Lastly, the percent gene expression for each GOI was calculated using the following formula: 2^-∆∆C(t) x100 = % Gene Expression. Since *apidaecin 1* gene has many repetitive elements that complicate qPCR primer design, we utilized primers developed by Evans et al. [22] for SYBR-green qPCR and modified thermocyling parameters (40 cycles of 95°C (30 s), 58°C (20 s), 72°C (60 s)); Cp = -3.7 +15.3, R^2^ =0.99. Melt curve analysis indicated a single predominant peak, but gel electrophoresis revealed faint smaller bands in addition to the expected 493 bp product; minor product priming likely contributed to increased standard error of the mean (SEM) values in this assay. 

### Honey bee gene expression microarray (sample preparation and hybridization)

To minimize variability between samples all arrayed bees were obtained from a single brood comb from a naturally-mated queen, therefore all the bees were age-matched half-sisters. The five representative bees from each condition (virus-infected, 3,750 PFU/bee; dsRNA-treated, 1 μg/bee; and mock-infected) were selected for microarray analysis were relatively pathogen free (assessed by APM and qPCR analysis, Table S1) [3]. To facilitate gene expression comparisons between multiple treatment groups we utilized a reference-design strategy in which each Cy5-labeled experimental sample was hybridized with a standardized Cy3-labeled reference sample. A complex RNA mixture representing hundreds of bees of various ages exposed to different treatment groups, served as the “reference RNA” sample.

Oligonucleotide-based microarrays (UIUC Honey Bee oligo 13K v1) were printed at the University of Illinois Keck Center for Comparative and Functional Genomics. Each microarray consisted of 28,000 features (14,400 unique features, including control spots, printed in duplicate on glass slides). The microarray has features representing 9,867 unique genes (8,902 with annotated *Drosophila* orthologs) [25], 2,729 EST probes, 22 probes specific for honey bee pathogens and control spots including 11 probes for green fluorescent protein (GFP) (Table S2) [27,29-32]. 

cDNA synthesis reactions were performed with SuperScriptIII (Invitrogen). In brief, RNA from five individual bees per treatment group (10 μg), oligo(dT)_20_ primers (10 μg) and random hexamer primers (500 ng) were combined in a 20 μL reaction volume, incubated at 65°C (5 min), and cooled on ice (1 min), and subsequently combined with 20 μL of 2X First-Strand Buffer containing SSIII (400 U), dNTPs (0.5 mM each dA/G/CTP; 0.2 mM dTTP; 0.3 mM aa-dUTP), DTT (5 mM), and RNaseOUT (80 U). Reverse transcription reactions were incubated for 12 hours at 42°C followed by inactivation of the reaction (70°C, 15 min). After cDNA synthesis, the RNA was hydrolyzed by NaOH (12 μL of 1N) and EDTA (12 μL of 0.5 M pH 8.0) treatment (65°C 15 min), neutralized by addition of HEPES pH 7 (60 μL). Amino-allyl cDNA was purified using MinElute columns (Qiagen). aa-cDNA (1750 ng) from each individual bee was labeled with Cy5 (sample) and reference RNA was labeled with Cy3. Dye labeling reactions [aa-cDNA (1750 ng) +16 nmoles dye in 20 μL buffer (0.1 NaOH pH 9) proceeded for 2 hours at RT in the dark, followed by MinElute column clean up and quantification. Hybridization A “reference RNA” strategy was utilized for comparative analysis of individual honey bee gene expression (5 individuals per group, hybridized independently). 70 μL of sample (containing Cy3-labeled sample and Cy3-labled reference; each normalized to 60 pmol dye) in hybridization buffer (25% formamide, 5x SSC, 0.1% SDS) was loaded under each lifterslip and arrays were hybridized at 42°C for 18 hours [33]. Post-hybridization washes were performed following removal of each lifter slip in wash #1 solution (2X SSC, 0.2% SDS), wash #1 for 10 minutes at 42°C; wash #2 (2X SSC, 0.2% SDS) for 10 minutes at 42°C; wash #3 (1X SSC) for 10 minutes; wash #4 (0.1X SSC) for 10 minutes. Slides were spun dry in an ozone-free hood. 

### Honey bee gene expression microarray analysis

Microarrays were scanned using an Axon 4B scanner and GenePix Pro software (Molecular Devices) for gridding and initial analysis. We performed global analysis of the differentially expressed genes (DEGs) using Bioconductor’s Linear Models for Microarray Analysis (LIMMA) [35] implemented in the R/Bioconductor platform [87]. In brief, microarrays were scanned and the data was loaded into the LIMMA program implemented in the R/Bioconductor platform. Fluorescent intensities were log_2_ transformed and corrected for the global median background signal using NORMEXP, followed by LOESS normalization within each array, and AQUATILE-SCALE normalization between arrays [35]. DUPCOR from LIMMA was used to correlate data from duplicate array features and the data was condensed into a single value for statistical analysis [16,19]. The data from the five individual arrays in each treatment group were analyzed in a pairwise comparison to five individual arrays from the mock-infected group (for details see R-code) using a contrast matrix. The data was fit to a linear model using EBAYES and false-discovery rate FDR p-value <0.05; an adjusted p-value <0.05 was selected to define significant differences between each experimental condition as compared to mock-infection. Experiments were designed to meet Minimum Information About a Microarray Experiment standards. All the protocols utilized for cDNA synthesis, labeling, hybridization, analysis (R-code for LIMMA) and all the raw microarray data obtained in this study was deposited at ArrayExpress [www.ebi.ac.uk/arrayexpress (accession no. E-MEXP-3608)]. In addition, the complete microarray expression data is provided in Excel format in Tables S4-S5. 

Gene Ontology annotations were based on *Drosophila melanogaster* orthologs identified for 8,902 of the 14,400 unique probes (62%) on the microarray (Tables S2-S4). Since ~25% of the genes on the array are represented by multiple probes, we used unique Entrez Gene identifiers to identify GO biological process (GOBPID) categories that were over-represented based on the number of regulated genes as compared to the number of genes on the honey bee genome microarray in each GO biological process category. Of the 8,902 microarray features with *Drosophila* orthologs, Ingenuity Pathway Analysis (IPA) (Ingenuity Systems, www.ingenuity.com) mapped 5,168 (~36% of the microarray features). Since some Entrez Gene IDs corresponded with multiple array features, there were actually 3,282 unique genes involved in characterized pathways. These 3,282 genes served as a background for over-representation analysis (ORA) using a false discovery rate (FDR) threshold of p-value < 0.05. Using these criteria, the number of DEGs analyzed for virus-infected bees was 62, whereas for dsRNA-treated bees it was 162.

## Supporting Information

Figure S1 **Honey bees are productively infected with a model virus, SINV-GFP.** (A) Increased GFP pixel counts in fluorescence microscope images of three bees each day post-infection demonstrated increased SINV-GFP infection over-time. Specifically, ImageJ was used to split the RGB layers into three images and GFP pixels above the threshold (50) were counted over a uniform area of each green image. GFP pixel counts on the injection day (day 0) and each day post-injection are presented. (B) Virus (SINV-GFP) spread from injection site (thorax) to the head (i.e., eye) (white arrow) demonstrated in this fluorescence microscope image. (C) RT-PCR detection of virus (SINV-GFP) (140 bp) in the head, thorax (injection site), and abdomen (ab) of bees that were infected with SINV-GFP containing lysates from other infected bees; bee 1 (lanes 1-3), bee 2 (lanes 4-6), uninfected bee (lane 7), and positive control PCR using the SINV-GFP containing plasmid as the template for PCR (lane 8). (D) SINV-GFP fluorescence (510-522 nm, relative arbitrary units) in individual lysates (5 bees per condition) from dissected honey bees over time; day post-injection, thorax (injection site), head and abdomen (distal sites).
(TIFF)

Figure S2 **Relative abundance of SINV-GFP in individual, dissected bees (5 per condition) was assessed by RT-qPCR.** Viral genome copy number per 500 ng RNA was calculated based on a SINV standard curve. Virus spread from the injection site (thorax 2.7x10^6^ + 4.1x10^4^ average genome copies per 500 ng RNA) to distal sites (head 3.9x10^5^+ 10.9x10^3^; abdomen 18.7x10^6^ + 2.3x10^5^ average genome copies per 500 ng) demonstrated productive infection of entire bee by day 3 post injection.
(TIFF)

Figure S3 **Western blot analysis of individual honey bees illustrates that the majority of bees (80%) demonstrated reduced virus in the presence of dsRNA whereas 20% were non-responsive to treatment.** Western blot analysis using α-GFP (top panel) and α-actin (bottom panel) antibodies demonstrated reduced SINV-GFP in individual, whole bee protein lysates of bees treated with dsRNA and poly(I:C), but not in bees treated with NTPs or dsDNA, as compared to bees inoculated with virus alone (lanes 2-6). ImageJ was used to quantify the pixels in equal sized GFP and actin regions of each lane in order to calculate the GFP to actin ratio in individual bee lysates. As expected, the mock-infected (mock, lane 1) bee had no detectable GFP, therefore the ratio the pixel counts within the GFP and actin regions of lane 1 was set to 1(*) to facilitate comparisons between lanes 2-31.
(EPS)

Table S1 **Pathogen test results for honey bees utilized for transcriptional analysis.** Arthropod Pathogen Microarray (APM) results determined that SINV-GFP was the predominant infection in virus-infected bees, whereas dsRNA-treated and mock-infected (buffer-injected) controls were not appreciably infected with common honey bee pathogens; APM oligonucleotide list (worksheet 1), APM results (worksheet 2). APM evaluation is performed using a computer algorithm called "Bee-Predict", described in Runckel* and Flenniken* et al. PLoS ONE 2011, but the raw APM data is provided in order to support our assessment that the predominant infection in virus-infected bees was SINV-GFP, and that all bees were not appreciably infected with other honey bee pathogens. Since there are multiple spots on the array for each pathogen (see oligo list) one positive spot is not sufficient for pathogen detection, often these single postive spots are located adjacent to a highly positive control spot and therefore are not valid detections.
(XLSX)

Table S2 **Honey Bee Gene Expression Microarray Annotation.**
(XLSX)

Table S3 **Complete Honey Bee Gene Expression Microarray Results.** Complete microarray results for virus-infected bees compared to mock-infected bees. Differentially expressed genes with adjusted p-value < 0.05 (worksheet 1) and all data (worksheet 2). Gene Ontology terms for biological process, molecular function, and cellular compartment were assigned and biological process assignments were further categorized into seven functional groups: immune response (red); transcription, splicing, rRNA processing and RNAi (blue); signaling (purple); trafficking (green); metabolism (yellow); translation and protein folding (orange); and chromatin regulation (teal). A small fraction of the DEGs (10-15%) belonged to other biological process gene ontologies (black) and a large fraction was not assigned (gray). Each pie chart represents 100% of the DEGs and gene numbers are reported in each section. *Each oligonucleotide pair was analyzed as a single gene and since multiple microarray probes corresponded to apidaecin variants the majority of annotated immune effectors in this Figure 5. (7 of 10 in virus-infected bees and 8 of 12 in dsRNA) correspond with apidaecin 1.
(XLSX)

Table S4 **Complete Honey Bee Gene Expression Microarray Results.** Complete microarray results for dsRNA-treated bees compared to mock-infected bees. Differentially expressed genes with adjusted p-value < 0.05 (worksheet 1) and all data (worksheet 2).
(XLSX)

Table S5 **Comparison of DEGs (adjusted p-value < 0.05) in virus-infected and dsRNA-treated honey bees using Venn diagram analysis (worksheet 1); virus only DEGs, RNA Only DEGs, and overlap between those two DEG lists.** We further examined overlapping DEG list, by sorting based on virus list fold-change (FC) (worksheet 2), dsRNA-treated list FC (worksheet3), virus list adjusted p-value (adj.p.val) (worksheet 4), and dsRNA-treated list adjusted p-value (worksheet 5).
(XLSX)

Table S6 **Pathways Over-Representation Analysis.** Over-representation analysis was performed on the DEGs involved in canonical pathways; the 3,282 unique genes on the array that are involved in characterized pathways served as a background and a false discovery rate (FDR) threshold of p-value < 0.05 was set. Using these criteria the number of differentially expressed (including both induced and reduced) genes analyzed virus-infected or dsRNA-treated as compared to mock treated controls was 62 and 162, respectively. Over-represented pathways for each experimental condition are listed as well as the p-value associated with each test.
(PDF)

Table S7 **Analysis of the expression of characterized insect immune genes in virus-infected and dsRNA-treated honey bees.**
(XLSX)

Table S8 **PCR and qPCR primer sequences.**
(XLSX)
