# Supplementary figures and images for: Non-Specific dsRNA-Mediated Antiviral Response in the Honey Bee
Source: PLoS One. 2013 Oct 10;8(10):e77263. doi: 10.1371/journal.pone.0077263 (PMC3795074; doi:10.1371/journal.pone.0077263)

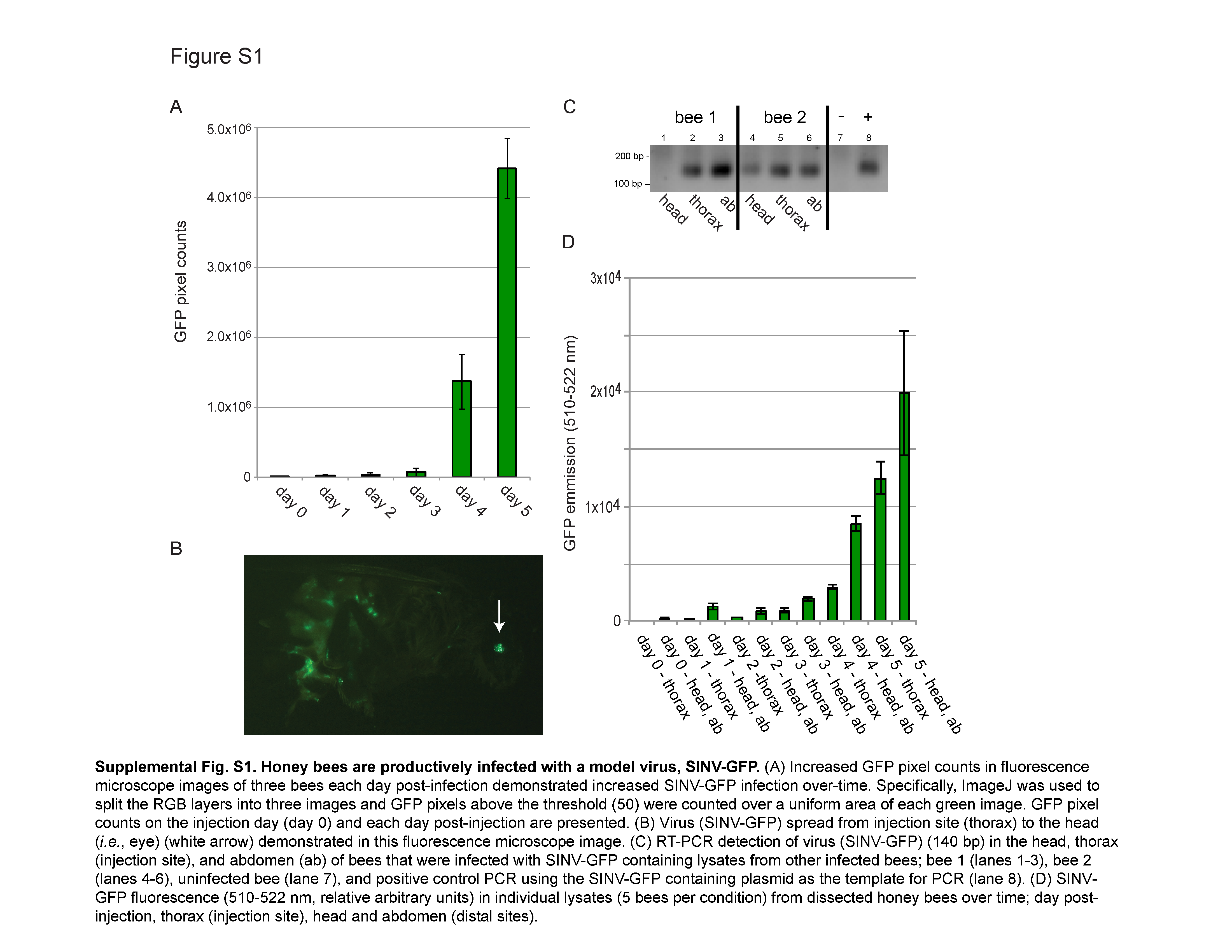

Supplement: Figure S1 — Honey bees are productively infected with a model virus, SINV-GFP. (A) Increased GFP pixel counts in fluorescence microscope images of three bees each day post-infection demonstrated increased SINV-GFP infection over-time. Specifically, ImageJ was used to split the RGB layers into three images and GFP pixels above the threshold (50) were counted over a uniform area of each green image. GFP pixel counts on the injection day (day 0) and each day post-injection are presented. (B) Virus (SINV-GFP) spread from injection site (thorax) to the head (i.e., eye) (white arrow) demonstrated in this fluorescence microscope image. (C) RT-PCR detection of virus (SINV-GFP) (140 bp) in the head, thorax (injection site), and abdomen (ab) of bees that were infected with SINV-GFP containing lysates from other infected bees; bee 1 (lanes 1-3), bee 2 (lanes 4-6), uninfected bee (lane 7), and positive control PCR using the SINV-GFP containing plasmid as the template for PCR (lane 8). (D) SINV-GFP fluorescence (510-522 nm, relative arbitrary units) in individual lysates (5 bees per condition) from dissected honey bees over time; day post-injection, thorax (injection site), head and abdomen (distal sites). (TIFF) [file pone.0077263.s001.tiff]

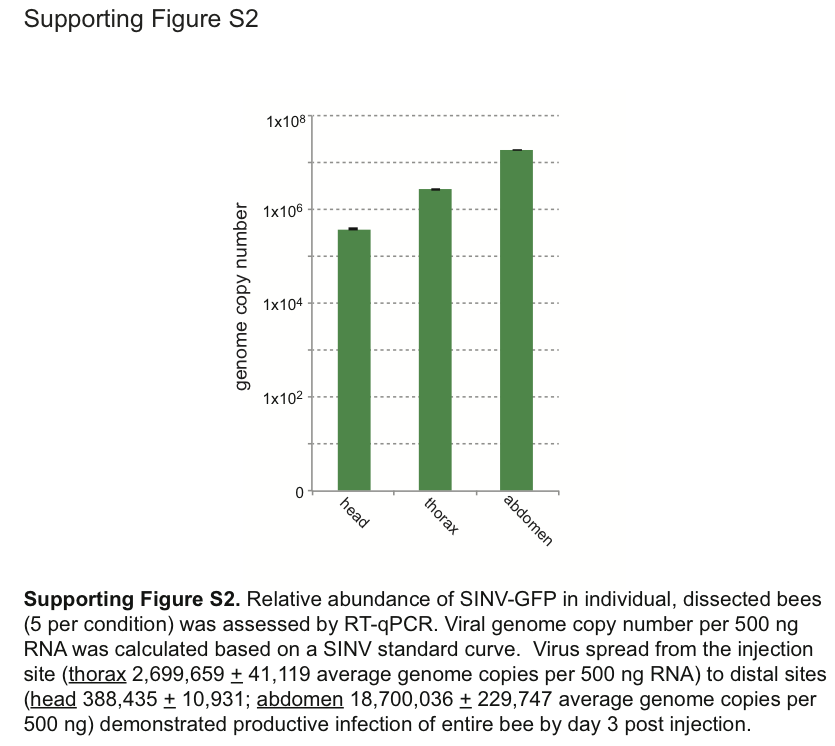

Supplement: Figure S2 — Relative abundance of SINV-GFP in individual, dissected bees (5 per condition) was assessed by RT-qPCR. Viral genome copy number per 500 ng RNA was calculated based on a SINV standard curve. Virus spread from the injection site (thorax 2.7x106 + 4.1x10^4^ average genome copies per 500 ng RNA) to distal sites (head 3.9x10^5^+ 10.9x103; abdomen 18.7x106 + 2.3x10^5^ average genome copies per 500 ng) demonstrated productive infection of entire bee by day 3 post injection. (TIFF) [file pone.0077263.s002.tiff]
