# Supplementary material for: Non-Specific dsRNA-Mediated Antiviral Response in the Honey Bee
Source: PLoS One. 2013 Oct 10;8(10):e77263. doi: 10.1371/journal.pone.0077263 (PMC3795074; doi:10.1371/journal.pone.0077263)
Supplement: Table S6 — Pathways Over-Representation Analysis. Over-representation analysis was performed on the DEGs involved in canonical pathways; the 3,282 unique genes on the array that are involved in characterized pathways served as a background and a false discovery rate (FDR) threshold of p-value < 0.05 was set. Using these criteria the number of differentially expressed (including both induced and reduced) genes analyzed virus-infected or dsRNA-treated as compared to mock treated controls was 62 and 162, respectively. Over-represented pathways for each experimental condition are listed as well as the p-value associated with each test. (PDF) [file pone.0077263.s009.pdf]

Supporting Table S6. Pathway Over-Representation Analysis

virus infected honey bees	
**Ingenuity Canonical Pathway**	**p-value**
Eicosanoid Signaling	0.0091
Glyoxylate and Dicarboxylate Metabolism	0.0204
Role of Oct4 in Mammalian Embryonic Stem Cell Pluripotency	0.0240
Serotonin Receptor Signaling	0.0240
Arachidonic Acid Metabolism	0.0355
Glutathione Metabolism	0.0490
**dsRNA treated honey bees**	
**Ingenuity Canonical Pathway**	**p-value**
Oxidative Phosphorylation	0.00005
Mitochondrial Dysfunction	0.00501
Glycosphingolipid Biosynthesis - Lactoseries	0.04898

Supporting Table S6.
Over-representation analysis was performed on the DEGs involved in canonical pathways; the 3,282 unique genes on the array that are involved in characterized pathways served as a background and a false discovery rate (FDR) threshold of p-value < 0.05 was set. Using these criteria the number of differentially expressed (including both induced and reduced) genes analyzed virus-infected or dsRNA-treated as compared to mock treated controls was 62 and 162, respectively. Over-represented pathways for each experimental condition are listed as well as the p-value associated with each test.
